# Three-Dimensional Surface Texture Analysis of Fluoride’s Effect on Enamel Erosion

**DOI:** 10.3390/jcm10194528

**Published:** 2021-09-30

**Authors:** Waseem Habashi, Amal Bader-Farraj, Nir Shpack, Ilan Beitlitum, Hila May, Rachel Sarig

**Affiliations:** 1 Department of Oral Biology, The Maurice and Gabriela Goldschleger School of Dental Medicine, Sackler Faculty of Medicine, Tel Aviv University, Tel Aviv 6997801, Israel; dr.waseemh@gmail.com (W.H.); dr.amalbader@gmail.com (A.B.-F.); 2 Department of Orthodontics, The Maurice and Gabriela Goldschleger School of Dental Medicine, Sackler Faculty of Medicine, Tel Aviv University, Tel Aviv 6997801, Israel; nir@shpack.co.il; 3 Department of Periodontology and Dental Implants, The Maurice and Gabriela Goldschleger School of Dental Medicine, Sackler Faculty of Medicine, Tel Aviv University, Tel Aviv 6997801, Israel; beilan@bezeqint.net; 4 The Shmunis Family Anthropology Institute, The Dan David Center for Human Evolution and Biohistory Research, Tel Aviv University, Tel Aviv 6997801, Israel; mayhila@tauex.tau.ac.il; 5 Department of Anatomy and Anthropology, Sackler Faculty of Medicine, Tel Aviv University, Tel Aviv 6997801, Israel

**Keywords:** dental erosion, enamel, fluoridation, texture surfaces

## Abstract

Enamel erosion has become a common clinical finding that often impairs dental esthetics and function. In the current study, we aimed to implement the three-dimensional surface texture analysis (3DST) method to explore the protective effect of fluoride on surface texture prior to erosive conditions. For each of the 50 teeth used in this study, the polished buccal enamel surface was divided into three separate areas: the first area was untouched polished enamel, the two other surfaces were immersed in 0.3% citric acid for 30 s. One was treated with high-concentration (19,000 ppm) sodium fluoride (NaF) solution prior to acid attack, and the other had no treatment prior to acid exposure. Enamel surface texture and step height measurements were obtained using a high-resolution disk scanning confocal microscope, and SEM images were also acquired. Surfaces treated with fluoride showed fewer variations in 3-D surface texture parameters than the eroded surface compared to the control group (*p* = 0.001). This was in accordance with the SEM descriptive images. The findings indicate that pre-fluoridated enamel areas were less affected by the acid and showed similar features to the untouched enamel. Moreover, a protective effect of the fluoride treatment against irreversible enamel damage was noted as the surface loss (step-height) was significantly reduced (*p* = 0.03). The study showed that 3DST analysis is a valuable methodology for detecting and quantifying subtle differences between the surfaces. When exploring the combination of all surface texture parameters, it was revealed that the pre-fluoridated eroded enamel surfaces showed considerable similarity to the untouched enamel.

## 1. Introduction

Dental erosion is associated with extrinsic or intrinsic acid that is not produced by bacteria. Intrinsic factors that may cause dental erosion include anorexia nervosa, bulimia nervosa, and conditions with frequent regurgitation of gastric acids, while extrinsic factors are mostly related with diet, environmental conditions, medications, and lifestyle [1]. In addition, the acid type and level, concentration of oral environmental calcium, phosphate, and fluoride, as well as the time and frequency of acid exposure, all play a role in the process of dental erosion [2]. Tooth erosion, if not managed through effective interventions, may result in substantial loss of dental tissue [2]. The damage to enamel can be either irreversible (i.e., total wiping out of dental hard tissue material) or in the form of partial tissue demineralization and softening [3], so both should be addressed in order to define the correct intervention.

Since enamel is a non-vital tissue and is not renewable, early prevention should be a priority before restorative management is considered. Preventive measures that address dental erosion can include dietary advice, stimulation of salivary flow rate, use of buffering medications, and optimization of fluoride regime [1,4]. Fluoride compounds were described to provide a positive effect on the inhibition and progression of dental erosion at high concentrations [5]. Many studies have investigated the effect of fluoride on the physical properties of enamel (i.e., microhardness, abrasion resistance) and its effect on surface changes, mainly by utilizing indentation methods and abrasion tests [6,7,8].

Quantitative methods commonly used to describe enamel roughness include profilometry [6,7,8,9], either contacting mechanical type—where a stylus sensor is used to contact the sample with light pressure [10]—or a non-contacting (laser scanning) type without any physical contact with the surface [11]. Surface topography can be examined using the line-profiling method (-2D roughness) or the areal-topography method (3-D roughness). The arithmetic average roughness parameter (Ra) is a popular 2-D roughness parameter used to analyze surface properties. Although it is sufficiently accurate in most cases, the advantage of 3-D roughness parameters is that they describe the nature of surface topography in finer details [12,13] and allow evaluation of the surface’s unevenness [14].

The usage of various 3-D technologies (e.g., scale-sensitive fractal analysis of high-resolution three-dimensional surface reconstructions) can provide an accurate and measurable characterization of surface texture [15,16]. Dental tissue surface texture analysis was utilized for various studies and provided new applications for clinical studies [15,17,18]. The three-dimensional surface texture (3DST) methodology is based on standardized parameters retrieved from engineering applications (ISO 25178-2 [19]), providing high-resolution measurable surface characterization. 

Another advantage of 3DST is that it enables highly accurate 3-D measurement in irregular surfaces (e.g., deep erosion pits, curved surfaces) [20]. The acquired parameters can provide data regarding height as well as spatial, hybrid, function, and segmentation features. Each group of parameters supply a different set of characteristics: Height parameters explore the statistical distribution along the *z*-axis, spatial parameters quantify the lateral directionality of the surface, hybrid parameters are a combination of texture amplitude and spacing measurements to characterize the overall nature of surface roughness, functional (volume) parameters indicate material void volume, and feature parameters characterize the projecting surface features (i.e., dales and hills) [13,18].

Therefore, the 3DST methodology can provide a new approach for exploring various experimental and clinical conditions. In the current study, we aimed to implement the 3DST methodology to explore the protective effect of fluoride on enamel surface texture prior to erosive conditions.

## 2. Materials and Methods

### 2.1. Study Sample

Fifty fully developed human permanent first and second lower molars were used in this study. All teeth were freshly extracted for periodontal reasons from male and female patients (age range 30–65 years). The study was approved by the ethics committee of Tel Aviv University (Authorization number: 0001117-1). Teeth with caries, restorations, or severe damage were excluded from the study. Once the tooth was extracted, the debris and soft tissue remnants were removed from the root with a sharp scalpel, then it was examined under proper light and magnification to detect any enamel anomalies, such as surface cracks, discoloration, or incipient caries. The teeth were repeatedly washed with double deionized water and then stored in 0.1% thymol solution [21]. 

### 2.2. Sample Preparation and Study Workflow

Original enamel surface shows great variability in its topography [22], thus making the polishing of the surface necessary for standardization purposes. The buccal surfaces were polished prior to analysis using polishing disks, 30 s each, as follows: coarse, medium, fine, and finally superfine aluminum oxide impregnated disks (Sof-Lex, 3M ESPE) using a low-speed headpiece under dry conditions. Ultra-sonication in double deionized water was then performed to remove any excess debris.

For each of the 50 teeth used in this study, the polished buccal enamel surface was divided into three separate areas by placing a borderline made of polyvinyl siloxane material (Express, 3M ESPE). The first area was untouched polished enamel (normal enamel—NE), the second was treated with highly concentrated (19,000 ppm) sodium fluoride solution for two minutes prior to acid attack (fluoridated eroded enamel—FEE), and the third had no fluoride treatment and was exposed to acid (eroded enamel—EE). Thorough rinsing and blot drying of the specimens was performed and the FEE and EE surfaces were immersed in 0.3% citric acid, pH 3.2 (adjusted using sodium hydroxide buffer [23]) for 30 s.

The fluoride solution was prepared by mixing 99% pure sodium fluoride (Sigma–Aldrich Company Ltd., Gillingham, UK) with double deionized water (pH 7.0) (21 g NaF/500 mL deionized water). Ion-selective fluoride electrode (Orion Star A214, Thermo Scientific, Beverly, MA, USA) was utilized to calculate and verify consistency across prepared solutions, after being calibrated before each measurement. All the specimens were left to dry overnight before data collection to ensure consistent scanning of all surfaces under the same scanning condition.

### 2.3. Enamel Erosion Evaluation

Enamel surface texture was imaged using a high-resolution disk scanning confocal measuring system (µsurf Explorer, NanoFocus AG, Oberhausen, Germany) equipped with a 160L lens (100× magnification) under a total magnification of 1000×. Surface images and texture data were collected using a spatial resolution of x, y = 0.31 µm and axial resolution of z = 0.003 µm. 

Prior to the measurement, all specimens were visually inspected under low magnification (100×) to identify the borders of the three areas of interest using the same measuring system. Consequently, high-resolution scans of 160 × 160 µm area each were conducted: polished normal enamel (NE), eroded area (EE), and fluoridated eroded area (FEE). 

Measurements were disregarded if surface area defects were found or if less than 98% of surface points were captured, wherein scans were repeated at a slightly different location. Surfaces were analyzed for several parameters (i.e., height, functional, spatial, hybrid, and feature) using µsoft analysis premium software (v. 8.0, NanoFocus AG; a derivative of Mountains Analysis software by Digital Surf, Besancon, France), and filtering operators were employed following ISO/FDIS 25178-2 [19]. 

For the evaluation of irreversible erosion of dental tissue, the step-height technique [24] was employed using the µsoft analysis premium software (v. 8.0, NanoFocus AG; a derivative of Mountains Analysis software by Digital Surf, Besancon, France) to explore all teeth. According to this approach, when evaluating two continuous flattened surfaces that have undergone some damage, the surface that is less resistant will lose more material. As a result, a step or shoulder will be formed between the two, allowing us to estimate the amount of material loss. In the current study, the high-resolution images of 160×160 µm area taken from the border between the adjacent surfaces were used for this purpose. Multiple single step height measurement profiles were automatically obtained and averaged by the software for the representation of the step area [25]. The step height between the surfaces of the EE and FEE areas to normal enamel was measured, revealing the amount of surface loss. In addition, descriptive images taken from 8 specimens were performed using SEM (JEOL JSM-IT100 PLUS) with gold–palladium coating.

### 2.4. Statistical Analysis

Statistical analysis of data from the 50 teeth was performed using GraphPad Prism software (v. 8.0, GraphPad Software, SanDiego, CA, USA), with the level of significance set to *p* = 0.05. The outliers were removed from the analysis using the ROUT method (Q = 1%) [26]. The variance in surface characteristics between the different surfaces was visualized via principal component analysis (PCA) using PAST software v.3.16 [27]. Kolmogorov-Smirnov tests were carried out to verify the normality of the measurement distributions. One-way analysis of variance (ANOVA) with post hoc (Tukey) and Kruskal-Wallis tests were used.

## 3. Results

### 3.1. Surface Texture Analysis

Based on the surface texture parameters as defined by the International Organization for Standardization (ISO/FDIS 25178-2), a clear distinction could be noted between the examined areas of the 50 teeth (Table 1). The results indicated that height features (i.e., arithmetical mean height (Sa), root-mean-square height (Sq), maximum pit height (Sv), and maximum height (Sz)) were the most significant in differentiating the three groups (*p* < 0.001). Maximum peak height (Sp) showed almost equal values for FEE and EE, both higher than NE (*p* < 0.001). The extreme peak height (Sxp) showed the highest value for the EE group, then FEE, and lowest for NE (*p* < 0.001). The NE and EE groups showed a negative skewness, while the FEE group had a positive value. 

The related volume parameters describing the voids (i.e., material volume of peaks (Vmp), material volume of the core at a given material ratio (Vmc), void volume of the core (Vvc), void volume of the valley at a given material ratio (Vvv)) indicated the smallest voids for the NE group, the largest for EE, and intermediate values for the FEE group (*p* < 0.001). Moreover, measuring parts of the surface including 5 or 10 voids or hills (five-point peak height five-point (S5p), five-point valley height arithmetic (S5v), 10-point height (S10z)) also showed similar results to the volume parameters for all three groups (*p* < 0.001). The dale area (Sda), hill area (Sha), as well as both the dale volume (Sdv) and hill volume (Shv), were found to be highest in the FEE group (*p* < 0.02).

Principal component analysis (PCA) was performed for the 30 surface texture parameters (for all teeth), to present multivariate results on a two-dimensional plot. The graph represents the overall differences between the groups, taking into account the contribution of all 30 parameters, which otherwise would not be feasible to view on a single chart. Therefore, the considerable overlap between the NE and FEE points indicates the similarity of the measured surfaces, thus implying the protective effect of the fluoridation on the enamel surface.

**Figure 1:**
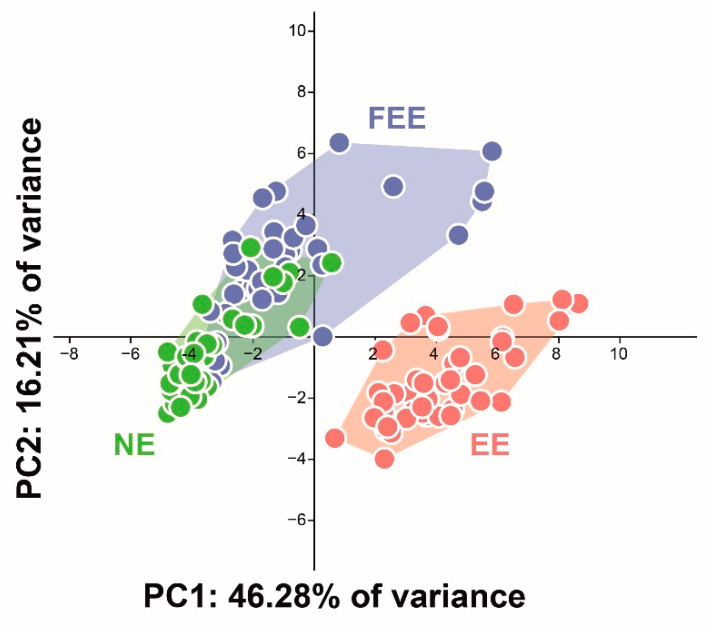
Principal component analysis (PCA): Graphic presentation of the overall differences between the groups, taking into account the contribution of all 30 parameters (NE is designated in green, EE in red, FEE blue).

The plot (Figure 1) shows a clear separation between the red (EE) group and the other two along the PC1 and PC2 axes (explaining 46% and 16% of variance, respectively).

### 3.2. Irreversible Erosion Evaluation 

**Figure 2:**
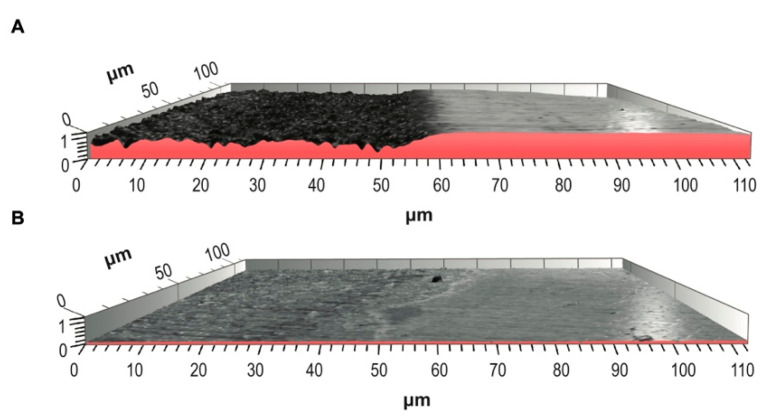
Three-Dimensional surface images of the discontinuity between the measured surfaces; (**A**) a comparison between NE (right) vs. EE (left). Note the material loss represented by a step between the surfaces. (**B**) the NE (right) vs. FEE (left). Here the difference between the surfaces is much less pronounced due to the protective effect of fluoride.

The discontinuity in surface representing the step height between NE and EE was distinguishably greater compared to the step between the NE and FEE (Figure 2). The fluoride pre-treated surface exhibited significantly (*p* = 0.03) lower irreversible enamel loss due to acid attack (0.39 ± 0.04 µm) compared to the control area (0.76 ± 0.05 µm) (Figure 2).

### 3.3. Descriptive Images

**Figure 3:**
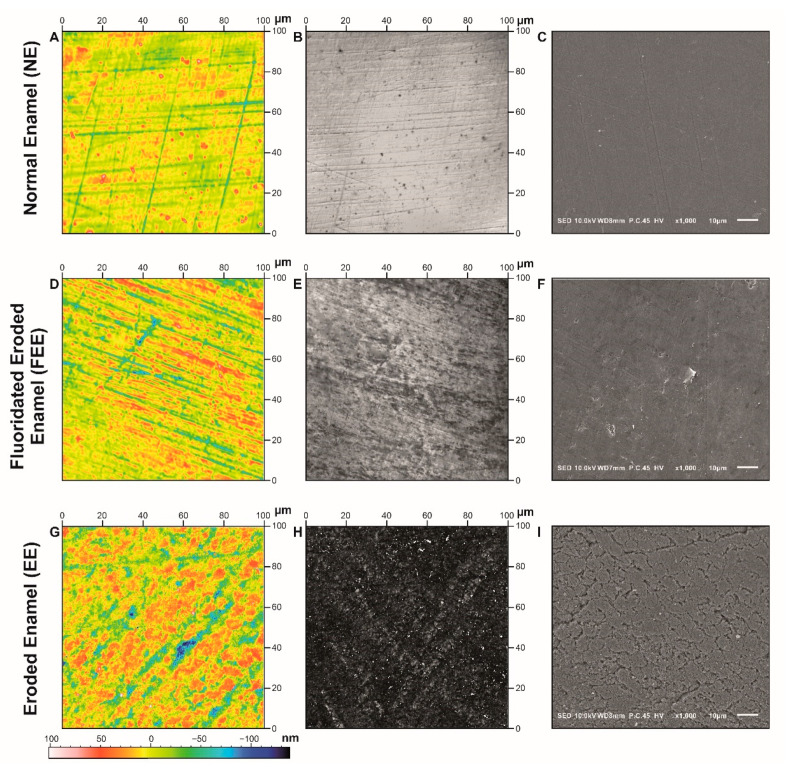
Representative images of enamel qualitative characteristics: NE group (**A**–**C**), FEE group (**D**–**F**), and EE group (**G**–**I**). The left column (**A**,**D**,**G**) presents the heights heat map; the middle column (**B**,**E**,**H**) presents the confocal microscopy photo simulation images; the right column (**C**,**F**,**I**) presents the SEM images. The EE group exhibited the greatest surface damage and notably rougher surface than the FEE and NE. This can be noted by the heat map images illustrating greater height differences (more red and blue vs. yellow). The darkness of the images in the middle column reflects the surface damage, as higher roughness induces light to disperse. The irregularity of the surfaces can be noted in the SEM images (right column).

Descriptive topographic images obtained by confocal microscopy imaging (of all teeth) and SEM images (eight teeth) were captured. This was done in order to examine the qualitative characteristics of the tested surface areas (Figure 3). 

Surface flatness is represented by a height heat map (Figure 3A,D,G). Homogenous colors indicating surface flatness can be seen in the normal enamel (Figure 3A), whereas contrasted colors indicate height differences (Figure 3G). Accordingly, the EE exhibited notable height surface differences compared to the other surfaces (NE and FEE). This is supported by the numerical height parameters where EE had the highest Sa value (0.126 µm), whereas the NE presented the lowest (0.024 µm) and FEE in between (0.041 µm). 

Exploring the confocal photo simulation images (Figure 3 middle column), the FEE appears darker than the NE group, yet not as dark as the EE. This may be explained by the surface being less reflective to the microscope’s light, due to a higher surface roughness level, causing light to disperse.

The SEM images (Figure 3C,F,I) clearly display the greatest surface damage for the EE, while the FEE exhibits some damage compared to NE but not as severe as EE. 

## 4. Discussion

In this study, we were able to provide a detailed quantitative and qualitative representation of the fluoridated, and normal enamel surfaces after short exposure to citric acid. 

The interaction of fluoride with the enamel can occur in several mechanisms depending on the concentration, agent used, time of application, and the pH of the environment [28]. It was suggested that the effect of the sodium fluoride high-concentration application is mainly through the formation of calcium fluoride (CaF_2_) and fluorapatite [29,30]. 

Many factors can impact the protective effect of fluoride-containing agents on dental erosion, such as excipients (thickeners, surfactants, stabilizers) and abrasives (type, size, shape, concentration, and abrasivity) [31,32,33]. In the current study, the topical vehicle was prepared as a pure form of sodium fluoride solution to allow easier rinsing with minimal residues to allow accurate evaluation of the surface.

It has been shown in a previous study that, under erosive challenges, two highly concentrated fluoride solutions (5000 ppm NaF and 19,000 ppm NaF) afforded significant protection to enamel from erosion compared to a low concentration solution [5].

The methodological parameters chosen for this study were based on the protocol suggested by Austin et al. [5], who showed that using high-concentration sodium fluoride treatment in a neutral pH environment provides a protective effect against erosion. The rationale in choosing a neutral environment was to keep the acidic attack similar to all samples, hence avoiding an acidic condition while applying the fluoride on the surface. A high concentration of fluoride was utilized to ensure that the protective effect will occur. 

The citric acid used in this study is commonly found in fruit juice drinks and most carbonated beverages. Reports suggest that children start consuming soft drinks at an increasingly younger age with higher intake frequencies [34]. Hence, the citric acid erosion hazard for the dental tissues is a growing concern for the dental field [35]. Toothpastes containing NaF can provide reasonable protection against erosion and may reduce enamel loss in cases of ordinary acid intakes [32]. Nevertheless, in erosion, the fluoride present on the enamel surface is likely to be readily dissolved due to the lower pH of the acids, thereby offering very limited protection [36].

Given the high sensitivity of the method used in this study, we were able to detect changes in the surface roughness and material loss using step height measurement after 30 s of acid exposure. It was previously shown that changes in surface texture can be noted as early as 10 s after exposure, yet step height changes were noted only 60 s after acidic exposure [8]. The significant differences in step height could be probably related to the large sample size we used (50 specimens compared to 10 specimens in the study by Mylonas et al. [8]).

The irreversibility of the material loss and the potential for aggravating this damage through abrasional interactions highlight the need for additional preventive measures. Moreover, in cases susceptible to high erosion conditions due to gastroesophageal reflux disease, eating disorders, or exaggerated dietary acid consumption [31,33], additional protective protocols should be considered. 

The values found in our study concur with previous observations that used the arithmetic average roughness parameter (Ra) [7,8,37,38], noting an increase in the Ra value after exposing enamel to an acidic environment and the protective effect of fluoride [39,40]. 

The protective effect of fluoride in maintaining the integrity of enamel from chemical breakdown in acidic environments is significant in the SEM images (Figure 3C,F,I) and well demonstrated by the texture analysis showing the high contrast between the NE and EE groups (Figure 1). The 3DST analysis provides a quantitative analysis to support the SEM images.

The advantage of 3DST is that it reveals the pattern of the surface modification. The height parameters analyzed in this study (i.e., Sa, Sq, Sv, Sz) exhibited significant differences between the three groups, indicating their potential for diagnosing the erosional and protective changes of the dental enamel surface [13]. We found these parameters to be highest in the EE group, indicating that surface erosion can be clearly represented by these features. Moreover, since normal enamel showed low height values, the increase in these parameters in case of erosion indicates the pattern of enamel loss at a micro scale. The FEE group showed less surface damage as the height values were lower than in the EE but still higher than NE. 

The same pattern was also noticed in the volume parameters (i.e., Vmp, Vmc, Vvc, Vvv), where the FEE group showed significantly lower values compared to the EE group. These findings clearly demonstrate the rationale behind etching enamel surfaces in restorative dentistry treatment to increase retention as larger core voids are correlated with increased micromechanical retention. Additionally, it explains the opposite rationale of not fluoridating the enamel surface prior to bonding procedures (e.g., bracket bonding) [41].

Common surface roughness measurements usually include the Sa (arithmetical mean height of a surface) or Ra (arithmetical mean height of a line). It expresses, as an absolute value, the difference in the height of each measured point compared to the arithmetical mean. This means that in case of a flat surface with a large void, for instance, the surface will present a high Sa value although most of it is flat. This measurement disregards the volume of the void and the diameter of the void as it only considers the height parameters. Considering only one value to explore the surface pattern cannot fully represent the texture of the surface.

In our study, the Sa parameter showed significant differences between the three groups (FEE, NE, and EE); however, when exploring the combination of all features (Figure 1), it was revealed that the FEE and NE surfaces showed considerable similarity in the measured surface texture properties.

The study showed that 3DST analysis is a valuable methodology as it allows detection and quantification of subtle differences between the surfaces. When exploring the combination of all surface texture parameters it was revealed that the pre-fluoridated eroded enamel surfaces showed considerable similarity to the untouched enamel.

## Figures and Tables

**Table 1:** Descriptive statistics of surface texture parameters (ISO 25178-2) are given for the 50 examined teeth (Mean, Standard deviation = SD), divided into the three experimental groups. *p*-value for comparisons resulting from the ANOVA are shown, and post hoc (Tukey) analysis uncover specific significant differences between the three groups.

Surface Texture Parameter				Polished Enamel (NE) (1)		Erosion Enamel (EE) (2)		Fluoridated/Erosion Enamel (FEE) (3)		Sig	Post Hoc Analysis:
			Units	Mean	SD	Mean	SD	Mean	SD		
Height	Root-mean-square height	Sq	µm	0.037	0.014	0.168	0.036	0.066	0.022	<0.001	1 ≠ 2 ≠ 3
	Skewness	Ssk		−0.841	1.607	−0.381	0.3656	0.040	2.198	<0.001	1 ≠ 3,2
	Kurtosis	Sku		15.67	11.5	4.39	1.15	22.38	19.13	0.003	1,3 ≠ 2
	Maximum peak height	Sp	µm	0.428	0.278	0.919	0.460	0.918	0.585	<0.001	2,3 ≠ 1
	Maximum pit height	Sv	µm	0.371	0.194	1.066	0.398	0.751	0.445	<0.001	1 ≠ 2 ≠ 3
	Maximum height	Sz	µm	0.889	0.514	2.041	0.753	1.813	0.992	<0.001	2,3 ≠ 1
	Arithmetic mean height	Sa	µm	0.024	0.007	0.126	0.025	0.041	0.010	<0.001	1 ≠ 2 ≠ 3
Functional (plane)	Areal material ratio	Smr	%	99.99	0.026	60.43	43.95	51.3	49.08	<0.001	2,3 ≠ 1
	Inverse areal material ratio	Smc	µm	0.031	0.008	0.195	0.039	0.058	0.013	<0.001	1 ≠ 2 ≠ 3
	Extreme peak height	Sxp	µm	0.082	0.029	0.362	0.101	0.130	0.036	<0.001	1 ≠ 2 ≠ 3
Spatial	Autocorrelation length	Sal	µm	3.684	1.196	3.825	1.194	5.827	1.741	<0.001	1,2 ≠ 3
	Texture-aspect ratio	Str		0.136	0.098	0.325	0.180	0.514	0.202	<0.001	1,2 ≠ 3
	Texture direction	Std	°	99.22	70.48	164.5	7.951	71.32	72.34	<0.001	1,3 ≠ 2
Hybrid	Root-mean-square gradient	Sdq		0.038	0.010	0.152	0.015	0.056	0.015	<0.001	1 ≠ 2 ≠ 3
	Developed interfacial area ratio	Sdr	%	0.067	0.029	1.15	0.219	0.148	0.062	<0.001	1 ≠ 2 ≠ 3
Functional (volume)	Material volume	Vm	µm^3^/µm^2^	0.002	0.001	0.007	0.001	0.004	0.001	<0.001	1 ≠ 2 ≠ 3
	Void volume	Vv	µm^3^/µm^2^	0.034	0.009	0.202	0.038	0.063	0.014	<0.001	1 ≠ 2 ≠ 3
	Peak material volume	Vmp	µm^3^/µm^2^	0.002	0.001	0.007	0.001	0.004	0.001	<0.001	1 ≠ 2 ≠ 3
	Core material volume	Vmc	µm^3^/µm^2^	0.022	0.005	0.136	0.028	0.041	0.008	<0.001	1 ≠ 2 ≠ 3
	Core void volume	Vvc	µm^3^/µm^2^	0.028	0.007	0.181	0.036	0.053	0.010	<0.001	1 ≠ 2 ≠ 3
	Pit void volume	Vvv	µm^3^/µm^2^	0.006	0.002	0.022	0.006	0.009	0.003	<0.001	1 ≠ 2 ≠ 3
Feature	Density of peaks	Spd	1/µm^2^	0.003	0.003	0.016	0.009	0.003	0.003	<0.001	1,3 ≠ 2
	Arithmetic mean peak curvature	Spc	1/µm	0.183	0.066	0.372	0.035	0.279	0.130	<0.001	1 ≠ 2 ≠ 3
	Ten point height	S10z	µm	0.353	0.129	1.258	0.335	0.743	0.320	<0.001	1 ≠ 2 ≠ 3
	Five point peak height	S5p	µm	0.174	0.088	0.490	0.138	0.418	0.238	<0.001	1 ≠ 2 ≠ 3
	Five point pit height	S5v	µm	0.201	0.096	0.742	0.240	0.298	0.107	<0.001	1 ≠ 2 ≠ 3
	Mean dale area	Sda	µm^2^	164.1	117	56.95	26.6	336	317.9	<0.001	1,3 ≠ 2
	Mean hill area	Sha	µm^2^	157.4	112.5	66.65	39.81	248.2	198.3	0.02	1 ≠ 2 ≠ 3
	Mean dale volume	Sdv	µm^3^	0.573	0.527	0.814	0.490	3.401	3.991	0.01	1 ≠ 2 ≠ 3
	Mean hill volume	Shv	µm^3^	0.693	0.606	1.046	0.710	2.118	1.990	0.02	1 ≠ 2 ≠ 3

## Data Availability

The data presented in this study are available on request from the corresponding author.
